# Impact of ALC Nadir and EDRIC on survival outcomes in limited-stage small cell lung cancer receiving chemoradiotherapy: a retrospective analysis comparing twice-daily and once-daily radiotherapy

**DOI:** 10.3389/fimmu.2026.1732515

**Published:** 2026-09-15

**Authors:** Jinquan Yao, Yikun Li, Yuxin Geng, Junhao Xu, Qixin Sun, Baiyang Huang, Bingwen Zou, Xiao Sun, Ning Tang, Feifei Teng

**Affiliations:** 1 Department of Radiation Oncology, Shandong Cancer Hospital and Institute, Shandong First Medical University and Shandong Academy of Medical Sciences, Jinan, China; 2 The First Affiliated Hospital of Shantou University Medical College, Shantou, China; 3 Department of Outpatient Chemotherapy, Harbin Medical University Cancer Hospital, Harbin, China; 4 School of Clinical Medicine, Shandong Second Medical University, Weifang, China; 5 Department of Radiation Oncology, West China Hospital of Sichuan University, Chengdu, China; 6 Department of Internal Medicine-Oncology, Shandong Cancer Hospital and Institute, Shandong First Medical University and Shandong Academy of Medical Sciences, Jinan, China

**Keywords:** absolute lymphocyte count, estimated dose of radiation to immune cells, immunotherapy, radiotherapy, small cell lung cancer

## Abstract

**Background:**

This study aimed to evaluate the prognostic value of absolute lymphocyte count (ALC) nadir and estimated dose of radiation to immune cells (EDRIC) in patients with limited-stage small cell lung cancer (LS-SCLC).

**Methods:**

This study analyzed 288 patients with LS-SCLC treated with chemoradiotherapy (CRT) between 2019 and 2023. ALC nadir and EDRIC were evaluated for prognostic value across patients receiving twice-daily (BID) and once-daily (QD) radiotherapy. Survival outcomes included overall survival (OS), progression-free survival (PFS), and brain metastasis-free survival (BMFS).

**Results:**

Despite lower lymphocyte counts during early treatment in the BID group, the ALC nadir was significantly higher in the BID group compared with the QD group (median ALC nadir: 0.60 **×** 10^9^/L vs. 0.49 **×** 10^9^/L, *P* < 0.001). In the BID group, patients with ALC nadir > 0.55 × 10^9^/L showed significantly longer OS (median OS: NR vs. 46.7 months, *P* = 0.011), PFS (*P* = 0.022), and BMFS (*P* = 0.013) compared with those with ALC nadir ≤ 0.55 × 10^9^/L. Patients in the high EDRIC group had significantly shorter PFS (*P* = 0.001) and BMFS (*P* = 0.003), and a trend toward shorter OS (*P* = 0.179). In the QD group, no significant differences were observed when stratified by ALC nadir or EDRIC (*P* > 0.05).

**Conclusion:**

ALC nadir and EDRIC demonstrated prognostic significance in the BID group but not in the QD group, suggesting potential differences in radiation-associated immune effects between fractionation schedules.

## 1 Introduction

Small cell lung cancer (SCLC) is an aggressive malignancy that originates from the neuroendocrine cells in the lungs. It is characterized by rapid growth, high invasiveness, early metastasis, and poor prognosis (1). Limited-stage small cell lung cancer (LS-SCLC) accounts for approximately one-third of all SCLC cases. For patients with LS-SCLC, chemoradiotherapy (CRT) remains the standard treatment. However, the 3-year overall survival (OS) rate remains relatively low, at approximately 56.5% (2). According to NCCN guidelines (3), patients with LS-SCLC are now advised to receive consolidation immunotherapy after completing CRT. The ADRIATIC study demonstrated that consolidation immunotherapy significantly prolonged both OS (median OS: 55.9 vs. 33.4 months, *P* = 0.01) and progression-free survival (PFS) (median PFS: 16.6 vs. 9.2 months, *P* = 0.02) *(*4). Despite the encouraging results from the ADRIATIC study, not all patients could derive benefit from consolidation immunotherapy, suggesting the importance of immune status during and after CRT. This highlights the need to identify patients who are suitable for receiving consolidation immunotherapy.

The immune system plays a critical role during treatment by facilitating the recognition and clearance of tumor cells, thereby contributing to improved therapeutic efficacy. Thoracic radiotherapy may stimulate the immune system by destroying tumor cells and promoting tumor antigen release (5). Nonetheless, it can also impair immune function by depleting lymphocytes (6). Previous studies have demonstrated that radiation-induced lymphopenia is significantly associated with poor prognosis across various solid tumors (6–9). Immune toxicity induced by radiotherapy may contribute significantly to poor clinical outcomes. Therefore, accurately quantifying the radiation dose delivered to immune-related structures is essential to elucidate the correlation with treatment outcomes. The estimated dose of radiation to immune cells (EDRIC) has emerged as a novel biomarker to quantify radiation-induced immune injury, incorporating both dose and target volume exposure. Yu et al. confirmed that EDRIC significantly correlates with absolute lymphocyte count (ALC) nadir and independently predicts OS and PFS in LS-SCLC patients (10).

Importantly, recent studies suggest that radiotherapy fractionation may influence the degree of immune suppression (11). The landmark Intergroup 0096 trial showed that twice-daily (BID) radiotherapy (45Gy/30F) significantly improved OS compared with once-daily (QD) radiotherapy (45Gy/25F), with an increase in median OS from 19 to 23 months (12). Nevertheless, the relatively lower biologically effective dose administered in the QD group raised concerns regarding the validity of this comparison. The more recent CONVERT and CALGB 30610 trials demonstrated comparable OS with ​BID radiotherapy (45Gy/30F) versus higher dose ​QD radiotherapy (66 Gy or 70 Gy) (13, 14). To date, limited studies have compared the immunologic effects of BID and QD radiotherapy in LS-SCLC. Furthermore, the predictive value of EDRIC and ALC nadir has not been comprehensively evaluated in relation to fractionation strategy and survival outcomes in this setting.

This study retrospectively analyzed patients with LS-SCLC receiving CRT, aiming to assess the correlation between EDRIC and ALC nadir and explore whether the prognostic values of these indicators for OS, PFS, and brain metastasis-free survival (BMFS) differ between the two fractionation schedules.

## 2 Methods

### 2.1 Patients

This retrospective study included patients with LS-SCLC who received CRT at Shandong Cancer Hospital and Institute between 2019 and 2023. Eligible patients were diagnosed with LS-SCLC according to the Veterans Administration Lung Study Group staging system. Eligible patients met the following criteria: (1) age ≥ 18 years; (2) receipt of at least 4 cycles of platinum-based chemotherapy; (3) received radiotherapy with either BID (45Gy/30F) or QD (60Gy/30F) fractionation; (4) availability of complete blood count (CBC) and follow-up data; (5) ALC prior to the initiation of thoracic radiotherapy ≥ 0.8 × 10^9^/L.

### 2.2 Treatment and follow-up

All patients underwent CRT, with a chemotherapy regimen consisting of etoposide plus either cisplatin or carboplatin. Thoracic radiotherapy was delivered using intensity-modulated radiotherapy and fractionation was either BID or QD. Some patients received prophylactic cranial irradiation (PCI) after CRT, based on clinical judgement and patient preference. During radiotherapy, complete blood counts were monitored weekly, and ALC values were collected. ALC nadir was defined as the lowest value recorded during thoracic radiotherapy. After completing treatment, patients were followed up every 3 months during the first year, every 6 months in the second year, and annually thereafter.

### 2.3 Calculation of EDRIC

EDRIC was used to estimate the radiation dose received by immune cells during thoracic radiotherapy. The calculation of EDRIC was based on previously published methods (15), considering mean lung dose (MLD), mean heart dose (MHD), mean body dose (MBD), and the number of radiotherapy fractions. The formula was:


$$\mathrm{EDRIC}\;=\;0.12\;\times \;\mathrm{MLD}\;+\;0.08\;\times \;\mathrm{MHD}+\;\left[0.45\;+\;0.35\;\times \;0.85\;\times \;{\left(\mathrm{number}\;\mathrm{of}\;\mathrm{fractions}/45\right)}^{1/2}\right]\;\times \;\mathrm{MBD}$$

### 2.4 Statistical analysis

OS was defined as the interval from diagnosis to death from any cause. PFS encompassed the period from diagnosis to either disease progression or death. BMFS was calculated from the start of diagnosis to radiologically confirmed brain metastases or death. Stratification into high and low groups was performed according to the median ALC nadir and EDRIC. Survival outcomes were evaluated using Kaplan–Meier analysis, with group comparisons performed via the log-rank test. Cox proportional hazards models were employed for both univariate and multivariate analyses to assess variables related to OS, PFS, and BMFS, identifying independent prognostic factors. All statistical analyses were two-sided, with *P* < 0.05 indicating statistical significance. Analyses were conducted using GraphPad Prism software version 10.1.1 and R software version 4.3.2.

## 3 Results

### 3.1 Baseline characteristics

A total of 288 patients with LS-SCLC who received CRT were retrospectively analyzed, of whom 145 received BID and 143 received QD radiotherapy (**Table 1**). **Figure 1** presents the flowchart of patient enrollment and exclusion. The median EDRIC was 5.61 Gy, and the median ALC nadir was 0.55 **×** 10^9^/L. When stratified by fractionation schedule, the median EDRIC was 4.60 Gy in the BID group and 6.70 Gy in the QD group. Despite lower lymphocyte counts during early treatment in the BID group, patients receiving BID radiotherapy had a significantly higher ALC nadir compared with those in the QD group (median: 0.60 **×** 10^9^/L vs. 0.49 **×** 10^9^/L, *P* < 0.001) (**Figure 2**). To explore the relationship between radiation exposure and lymphocyte suppression, we evaluated the correlation between EDRIC and ALC nadir. There was a significant inverse correlation between EDRIC and ALC nadir (R = –0.44, *P* < 0.0001) **Supplementary Figure 1**. This finding suggested that greater radiation exposure may lead to deeper lymphocyte depletion.

**Table 1:** Patients clinicopathological characteristics of the whole cohort.

Characteristics	BID (n=145)	QD (n=143)
Sex: no. (%)		
Male	101 (69.66)	103 (72.03)
Female	44 (30.34)	40 (27.97)
Age: no. (%)		
≤60	65 (44.83)	76 (53.15)
>60	80 (55.17)	67 (46.85)
Smoking status: no. (%)		
Never	84 (57.93)	74 (51.75)
Former or current	61 (42.07)	69 (48.25)
Location: no. (%)		
Right lung	78 (53.79)	74 (51.75)
Left lung	67 (46.21)	69 (48.25)
T stage: no. (%)		
≤2	86 (59.31)	83 (58.04)
>2	59 (40.69)	60 (41.96)
N stage: no. (%)		
≤1	29 (20.00)	31 (21.68)
>1	116 (80.00)	112 (78.32)
Induction chemotherapy: no. (%)		
No	27 (18.62)	21 (14.69)
Yes	118 (81.38)	122 (85.31)
Chemotherapy cycles: no. (%)		
≤4	51 (35.17)	44 (30.77)
>4	94 (64.83)	99 (69.23)
Chemotherapy regimen: no. (%)		
EP	73 (50.34)	60 (41.96)
EC	72 (49.66)	83 (58.04)
The response to CRT: no. (%)		
CR	15 (10.34)	5 (3.50)
PR	114 (78.62)	112 (78.32)
SD	16 (11.03)	26 (18.18)
PCI: no. (%)		
No	79 (54.48)	68 (47.55)
Yes	66 (45.52)	75 (52.45)
ALC nadir: no. (%)		
≤0.55	57 (39.31)	93 (65.03)
>0.55	88 (60.69)	50 (34.97)
EDRIC: no. (%)		
≤5.61	100 (68.97)	44 (30.77)
>5.61	45 (31.03)	99 (69.23)

EP, Etoposide plus cisplatin; EC, Etoposide plus carboplatin; CRT, Chemoradiotherapy; CR, Complete response; PR, Partial response; SD, Stable disease; PCI, Prophylactic cranial irradiation; ALC, Absolute lymphocyte count; EDRIC, Estimated dose of radiation to immune cells; BID, Twice-daily; QD, Once-daily.

**Figure 1:**
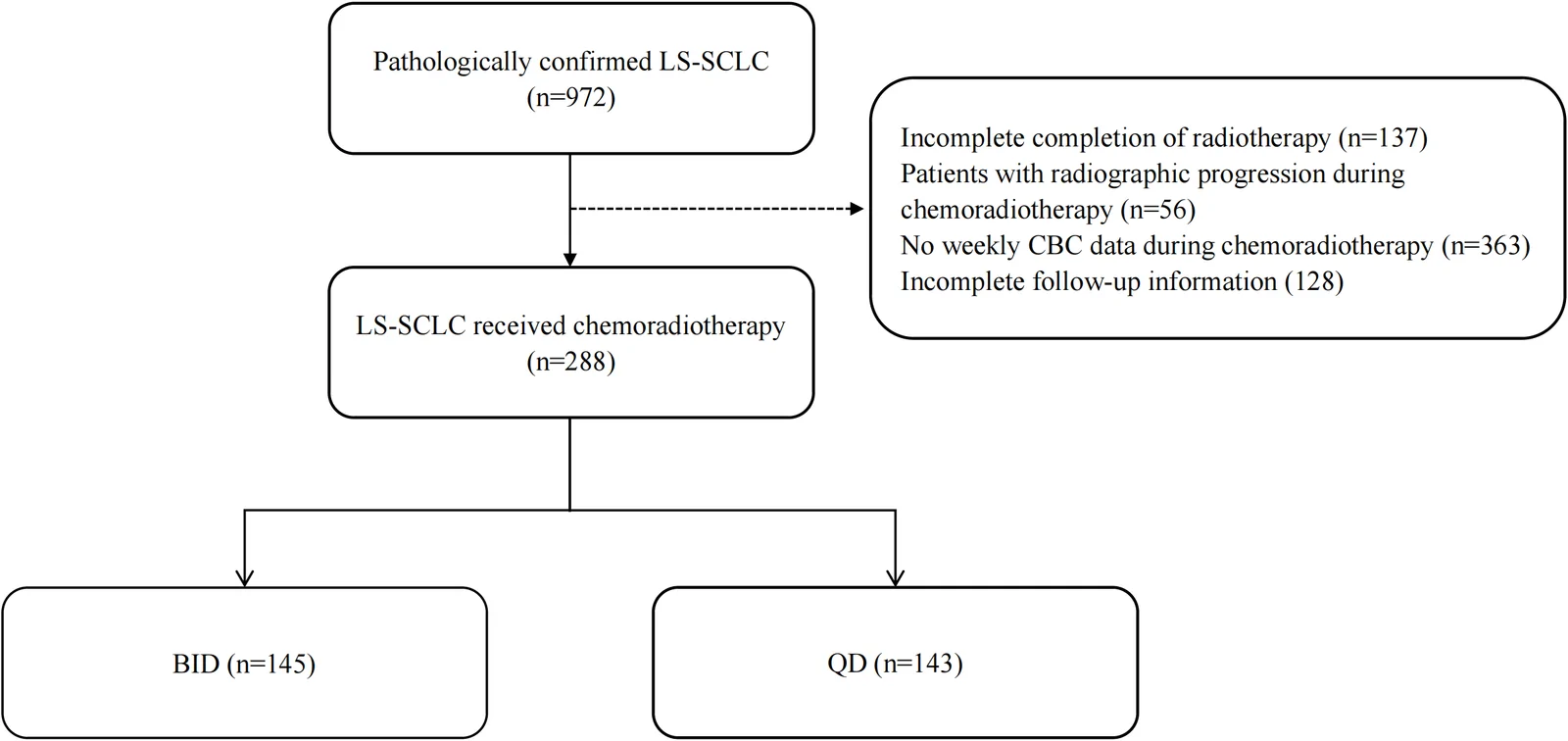
Patient enrollment and exclusion flowchart.

**Figure 2:**
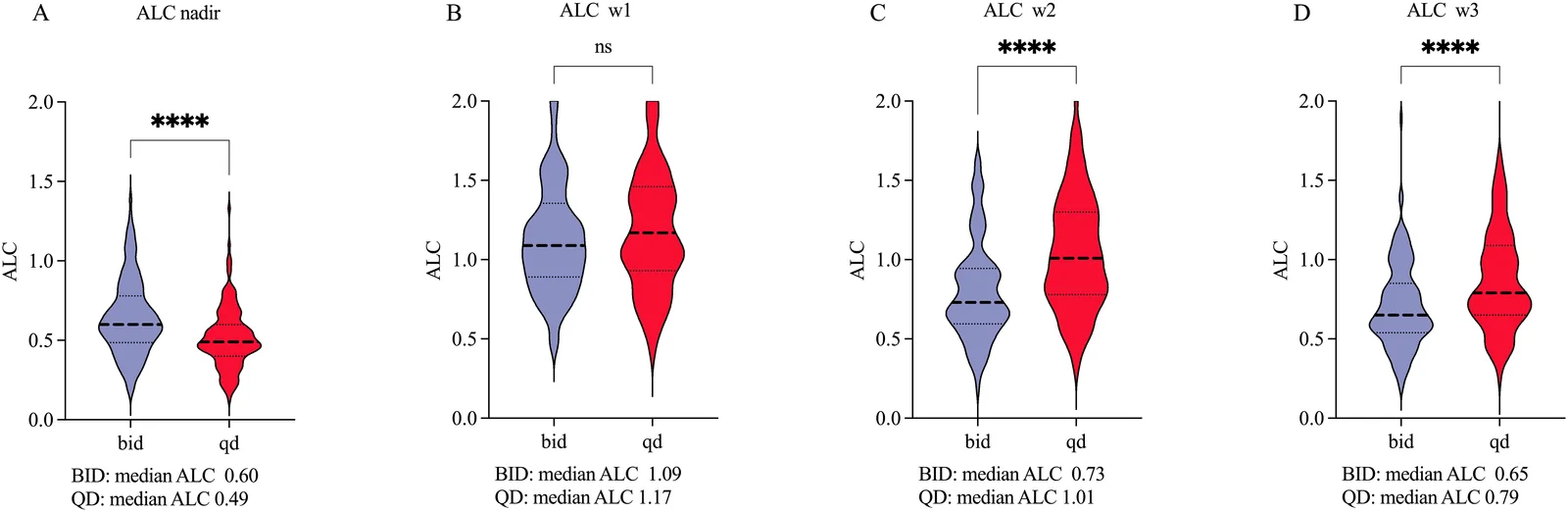
Comparison of ALC between BID and QD groups. **(A)** ALC nadir during radiotherapy. **(B)** ALC at Week 1. **(C)** ALC at Week 2. **(D)** ALC at Week 3. ALC, Absolute lymphocyte count; BID, Twice-daily; QD, Once-daily. ****P < 0.0001; ns, not significant.

### 3.2 Survival outcomes

The median OS, BMFS, and PFS in all patients were NR, 32.6 months, and 14.2 months, respectively (**Figure 3A**). When stratified by ALC nadir, statistically significant differences were observed in OS (*P* = 0.021) (**Figure 3B**), but there were no significant differences in PFS (*P* = 0.107) (**Figure 3C**) or BMFS (*P* = 0.32) (**Figure 3D**). In the BID group, patients with an ALC nadir > 0.55 × 10^9^/L had significantly longer OS than those with an ALC nadir ≤ 0.55 × 10^9^/L (median OS: NR vs. 46.7 months, *P* = 0.011) (**Figure 4A**). Similarly, there were significant differences in PFS (median PFS: 17.2 vs. 11.6 months, *P* = 0.022) (**Figure 4C**) and BMFS (median BMFS: NR vs. 25.7 months, *P* = 0.013) (**Figure 4E**). Based on the median EDRIC, patients were stratified into high and low EDRIC groups for further survival analyses. Patients with an EDRIC > 5.61 Gy had shorter OS compared with those with an EDRIC ≤ 5.61 Gy, although the difference was not statistically significant (**Figure 4B**). Significant differences were observed in PFS (median PFS: 10.2 vs. 18.0 months, *P* = 0.001) (**Figure 4D**) and BMFS (median BMFS: 16.2 vs. NR, *P* = 0.003) (**Figure 4F**) between patients with an EDRIC > 5.61 Gy and those with an EDRIC ≤ 5.61 Gy.

**Figure 3:**
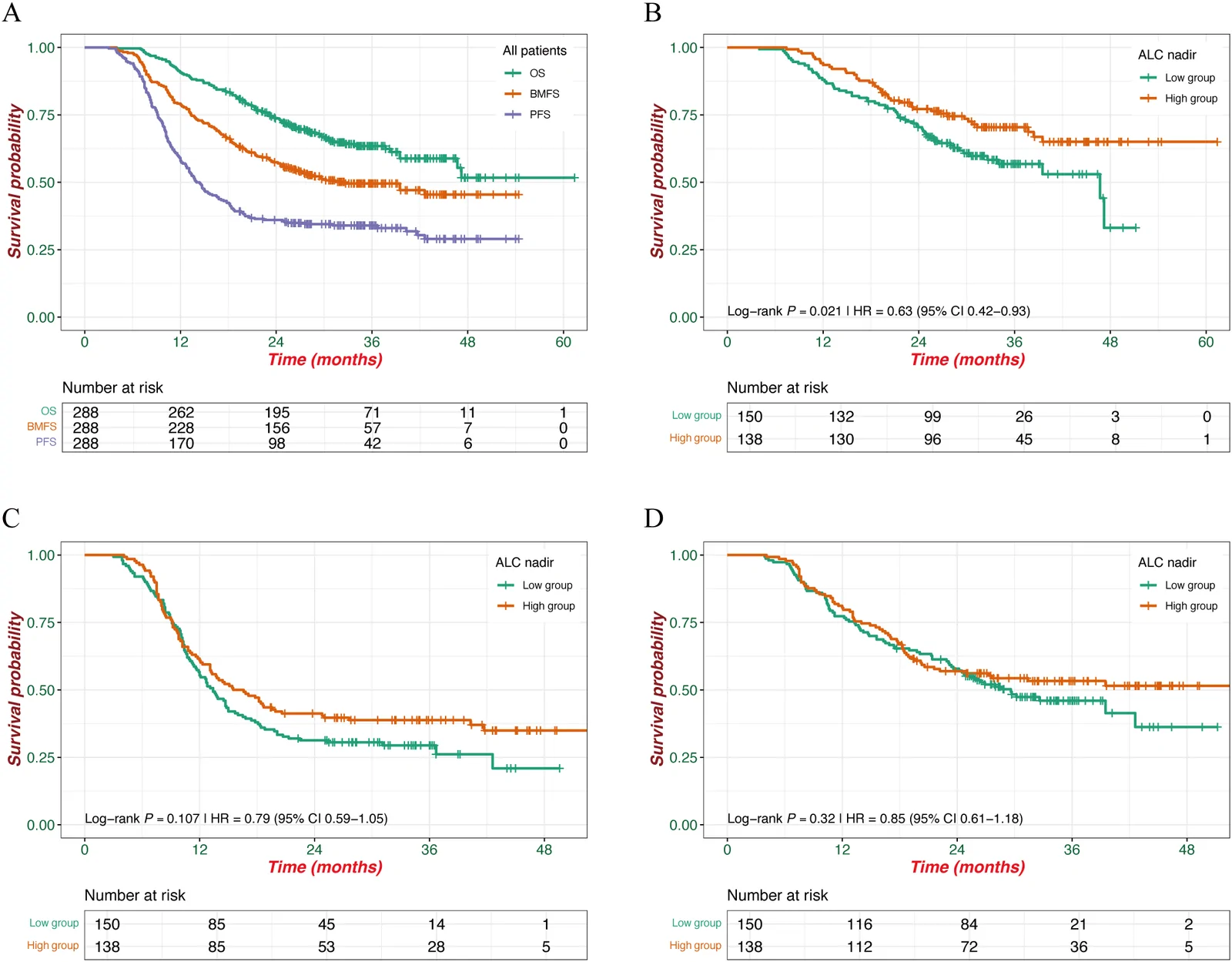
Kaplan–Meier survival curves for all patients. **(A)** OS, PFS, and BMFS for all patients. **(B)** OS for all patients stratified by median ALC nadir. **(C)** PFS for all patients stratified by median ALC nadir. **(D)** BMFS for all patients stratified by median ALC nadir. OS, Overall survival; PFS, Progression-free survival; BMFS, Brain metastasis-free survival; ALC, Absolute lymphocyte count.

**Figure 4:**
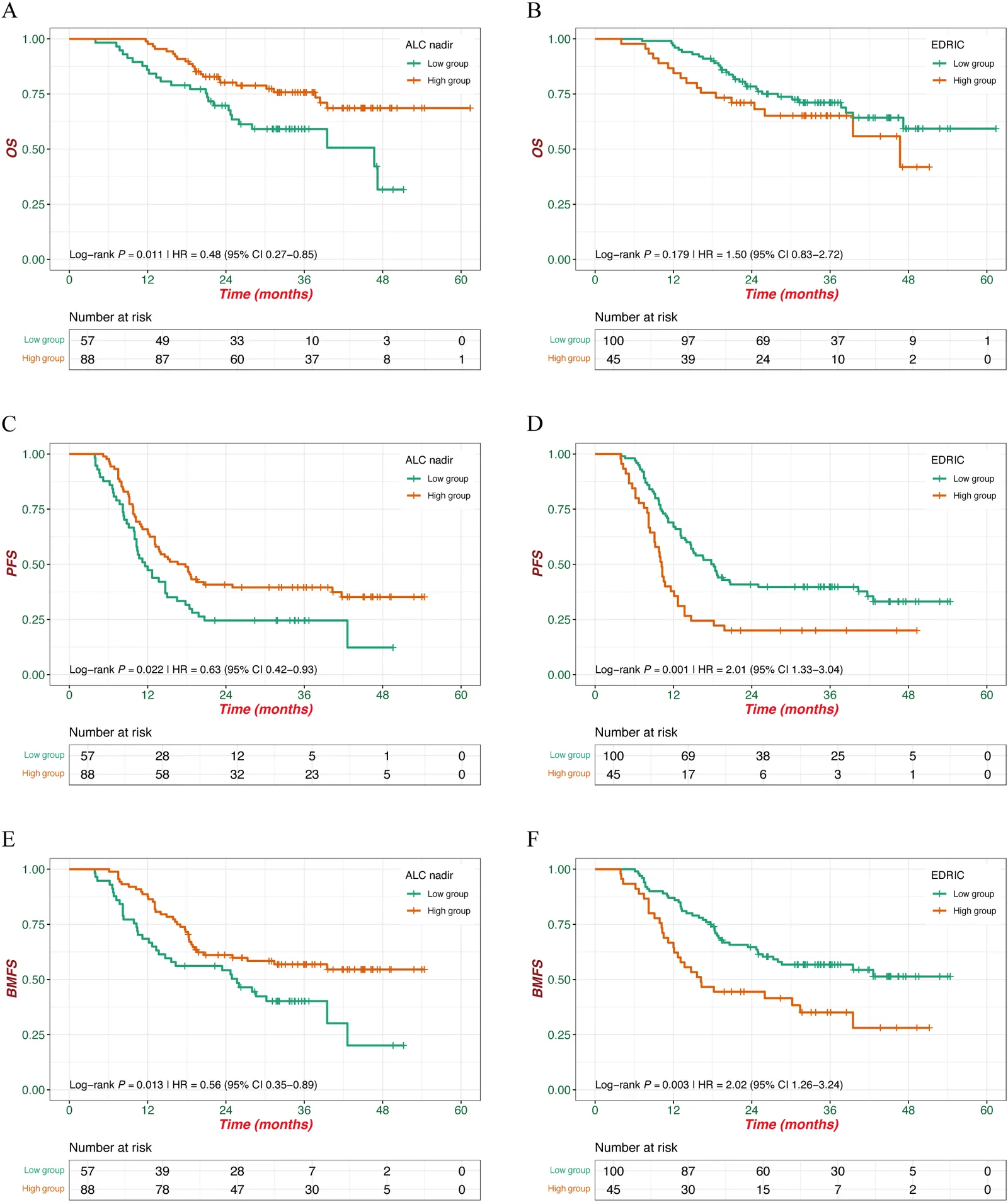
Kaplan–Meier survival curves in the BID group stratified by median ALC nadir and EDRIC. **(A, C, E)** Survival outcomes stratified by median ALC nadir: OS **(A)**, PFS **(C)**, and BMFS **(E)**. **(B, D, F)** Survival outcomes stratified by median EDRIC: OS **(B)**, PFS **(D)**, and BMFS **(F)**. ALC, Absolute lymphocyte count; BID, Twice-daily; OS, Overall survival; PFS, Progression-free survival; BMFS, Brain metastasis-free survival.

In the QD group, there were no significant differences in OS (median OS: NR vs. NR, *P* = 0.688) (**Figure 5A**), PFS (median PFS: 14.9 vs. 13.9 months, *P* = 0.854) (**Figure 5C**), or BMFS (median BMFS: 24.9 vs. 32.6 months, *P* = 0.329) (**Figure 5E**) between patients with an ALC nadir > 0.55 × 10^9^/L and those with an ALC nadir ≤ 0.55 × 10^9^/L. Based on the median EDRIC, no significant difference in OS was observed between the high and low groups (median OS: NR vs. NR, *P* = 0.778) (**Figure 5B**). Similarly, PFS did not differ significantly between the groups (median PFS: 13.9 vs. 14.9 months, *P* = 0.786) (**Figure 5D**). BMFS also showed no significant difference between the two groups (median BMFS: NR vs. 26.9 months, *P* = 0.431) (**Figure 5F**). To assess the robustness of the findings, a sensitivity analysis was conducted using median cutoffs derived independently within the QD group. This analysis yielded results consistent with the primary analysis, with no statistically significant differences observed in OS, PFS, or BMFS (**Supplementary Figure 2**).

**Figure 5:**
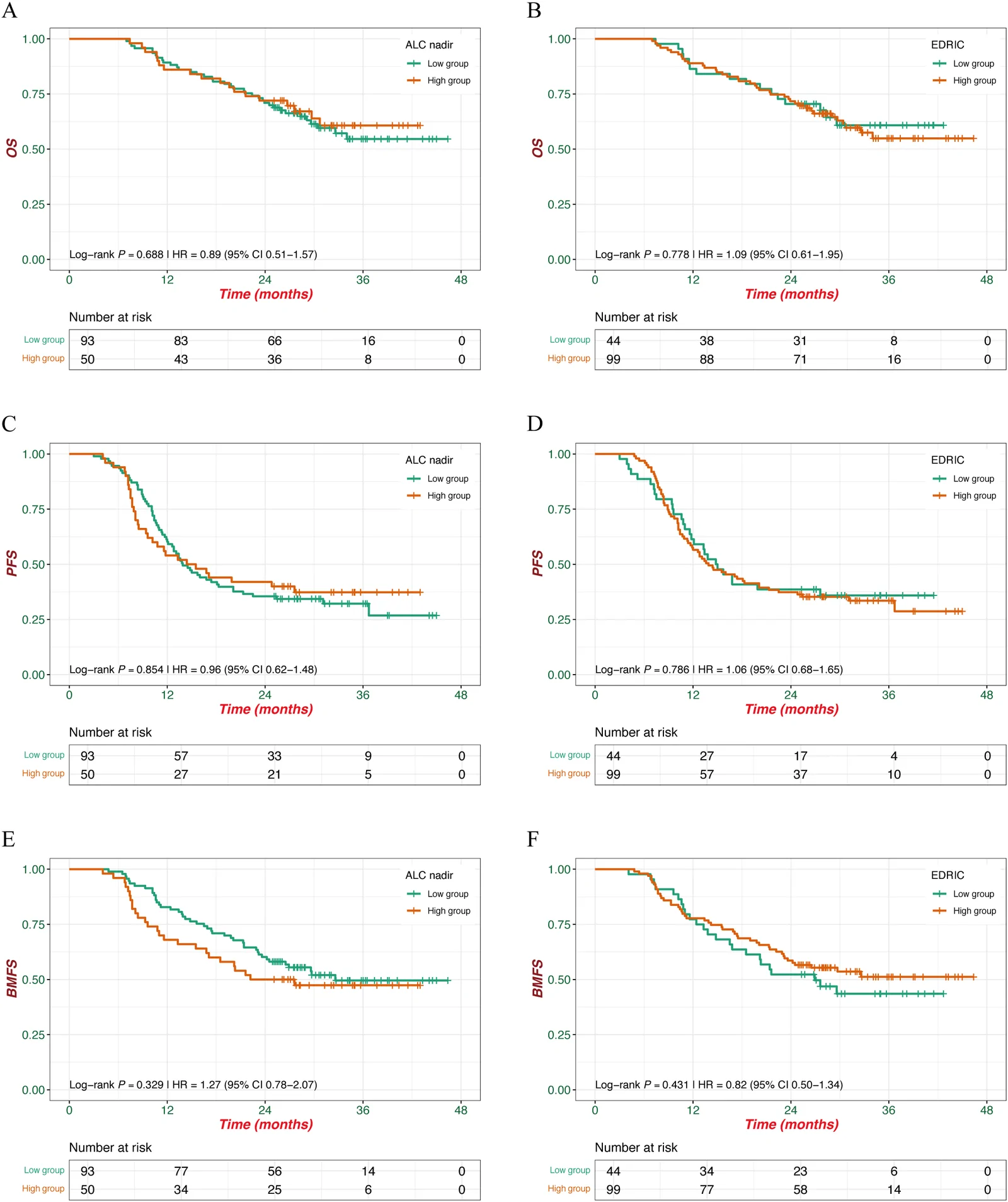
Kaplan–Meier survival curves in the QD group stratified by median ALC nadir and EDRIC. **(A, C, E)** Survival outcomes stratified by median ALC nadir: OS **(A)**, PFS **(C)**, and BMFS **(E)**. **(B, D, F)** Survival outcomes stratified by median EDRIC: OS **(B)**, PFS **(D)**, and BMFS **(F)**. ALC, Absolute lymphocyte count; OS, Overall survival; PFS, Progression-free survival; BMFS, Brain metastasis-free survival.

### 3.3 Univariate and multivariate analyses of OS, PFS, and BMFS in the BID group

Univariate analysis showed that sex (*P* = 0.069), age (*P* = 0.033), smoking status (*P* = 0.063), PCI (*P* = 0.005), and ALC nadir (*P* = 0.011) were associated with OS. Multivariate analysis identified PCI (*P* = 0.010) and ALC nadir (*P* = 0.008) as independent prognostic factors for OS (**Table 2**). For PFS, multivariate analysis indicated that PCI (*P* < 0.001) and EDRIC (*P* = 0.003) were independent prognostic factors (**Table 3**). For BMFS, PCI (*P* < 0.001) and EDRIC (*P* = 0.011) remained independent prognostic factors **Table 4**.

**Table 2:** Univariate and multivariate analyses of the OS in BID group.

Characteristics	Univariate analysis		Multivariate analysis	
	HR (95%CI)	P-value	HR (95%CI)	P-value
Sex				
Male	1		1	
Female	0.52 (0.26 ~ 1.05)	**0.069**	0.63 (0.28 ~ 1.44)	0.274
Age				
≤60	1		1	
>60	1.94 (1.05 ~ 3.58)	**0.033**	1.77 (0.95 ~ 3.28)	0.070
Smoking status				
Never	1		1	
Former or current	1.72 (0.97 ~ 3.03)	0.063	1.38 (0.70 ~ 2.71)	0.346
Location				
Right lung	1			
Left lung	1.25 (0.71 ~ 2.20)	0.447		
T stage				
≤2	1			
>2	0.95 (0.53 ~ 1.69)	0.855		
N stage				
≤1	1			
>1	1.18 (0.55 ~ 2.52)	0.668		
Induction chemotherapy				
No	1			
Yes	1.01 (0.45 ~ 2.26)	0.983		
Chemotherapy cycles				
≤4	1			
>4	0.68 (0.38 ~ 1.22)	0.198		
Chemotherapy regimen				
EP	1			
EC	1.05 (0.60 ~ 1.86)	0.864		
PCI				
No	1		1	
Yes	0.41 (0.22 ~ 0.76)	**0.005**	0.44 (0.23 ~ 0.82)	**0.010**
ALC nadir				
≤0.55	1		1	
>0.55	0.48 (0.27 ~ 0.85)	**0.011**	0.46 (0.26 ~ 0.82)	**0.008**
EDRIC				
≤5.61	1			
>5.61	1.50 (0.83 ~ 2.72)	0.180		

OS, Overall survival; BID, Twice-daily; EP, Etoposide plus cisplatin; EC, Etoposide plus carboplatin; PCI, Prophylactic cranial irradiation; ALC, Absolute lymphocyte count; EDRIC, Estimated dose of radiation to immune cells.

Bold values indicate statistical significance, defined as P < 0.05.

**Table 3:** Univariate and multivariate analyses of the PFS in BID group.

Characteristics	Univariate analysis		Multivariate analysis	
	HR (95%CI)	P-value	HR (95%CI)	P-value
Sex				
Male	1			
Female	0.77 (0.50 ~ 1.20)	0.250		
Age				
≤60	1			
>60	1.32 (0.88 ~ 1.96)	0.176		
Smoking status				
Never	1			
Former or current	1.20 (0.81 ~ 1.79)	0.361		
Location				
Right lung	1			
Left lung	1.06 (0.72 ~ 1.58)	0.756		
T stage				
≤2	1			
>2	0.91 (0.61 ~ 1.37)	0.654		
N stage				
≤1	1			
>1	1.15 (0.69 ~ 1.92)	0.587		
Induction chemotherapy				
No	1			
Yes	0.88 (0.53 ~ 1.47)	0.632		
Chemotherapy cycles				
≤4	1			
>4	0.86 (0.57 ~ 1.30)	0.462		
Chemotherapy regimen				
EP	1			
EC	0.90 (0.61 ~ 1.34)	0.604		
PCI				
No	1		1	
Yes	0.39 (0.26 ~ 0.59)	**<.001**	0.39 (0.26 ~ 0.60)	**<.001**
ALC nadir				
≤0.55	1		1	
>0.55	0.63 (0.42 ~ 0.93)	**0.022**	0.87 (0.57 ~ 1.34)	0.528
EDRIC				
≤5.61	1		1	
>5.61	2.00 (1.32 ~ 3.03)	**<.001**	1.94 (1.25 ~ 3.01)	**0.003**

PFS, Progression-free survival; BID, Twice-daily; EP, Etoposide plus cisplatin; EC, Etoposide plus carboplatin; PCI, Prophylactic cranial irradiation; ALC, Absolute lymphocyte count; EDRIC, Estimated dose of radiation to immune cells.

Bold values indicate statistical significance, defined as P < 0.05.

**Table 4:** Univariate and multivariate analyses of the BMFS in BID group.

Characteristics	Univariate analysis		Multivariate analysis	
	HR (95%CI)	P-value	HR (95%CI)	P-value
Sex				
Male	1			
Female	0.72 (0.42 ~ 1.21)	0.211		
Age				
≤60	1			
>60	1.36 (0.85 ~ 2.18)	0.198		
Smoking status				
Never	1			
Former or current	1.38 (0.87 ~ 2.19)	0.170		
Location				
Right lung	1			
Left lung	1.02 (0.64 ~ 1.62)	0.934		
T stage				
≤2	1			
>2	0.88 (0.55 ~ 1.41)	0.595		
N stage				
≤1	1			
>1	1.23 (0.68 ~ 2.24)	0.495		
Induction chemotherapy				
No	1			
Yes	1.02 (0.55 ~ 1.90)	0.942		
Chemotherapy cycles				
≤4	1			
>4	1.06 (0.64 ~ 1.73)	0.827		
Chemotherapy regimen				
EP	1			
EC	0.83 (0.53 ~ 1.32)	0.437		
PCI				
No	1		1	
Yes	0.37 (0.22 ~ 0.60)	**<.001**	0.37 (0.23 ~ 0.62)	**<0.001**
ALC nadir				
≤0.55	1		1	
>0.55	0.56 (0.35 ~ 0.89)	**0.013**	0.70 (0.44 ~ 1.13)	0.145
EDRIC				
≤5.61	1		1	
>5.61	2.02 (1.26 ~ 3.23)	**0.004**	1.88 (1.16 ~ 3.06)	**0.011**

BMFS, Brain Metastasis-free survival; BID, Twice-daily; EP, Etoposide plus cisplatin; EC, Etoposide plus carboplatin; PCI, Prophylactic cranial irradiation; ALC, Absolute lymphocyte count; EDRIC, Estimated dose of radiation to immune cells.

Bold values indicate statistical significance, defined as P < 0.05.

## 4 Discussion

In this study, we retrospectively analyzed the prognostic significance of two immune-related biomarkers in patients with LS-SCLC receiving CRT, with particular attention to differences between BID and QD fractionation schedules. Our findings demonstrate that both ALC nadir and EDRIC were significantly associated with survival outcomes in the BID group but not in the QD group. These results suggest that the prognostic relevance of immune-related biomarkers may vary according to the radiotherapy fractionation schedule.

Lymphocytes, as a key component of the peripheral immune system, are associated with tumor initiation and progression, exhibiting high sensitivity to radiotherapy, with their counts significantly decreasing during radiotherapy. Prior studies have shown that lymphocyte loss during radiotherapy is a surrogate for immune injury and is linked to poor survival outcomes (10, 16–19). Interestingly, despite lower lymphocyte counts during early treatment in the BID group, the ALC nadir was significantly higher in the BID group compared with the QD group in our study (median ALC nadir: 0.60 **×** 10^9^/L vs. 0.49 **×** 10^9^/L, *P* < 0.001), implying that the early reduction and the depth of nadir may represent distinct biological processes with potentially different implications for immune recovery and prognosis. The early reduction in lymphocytes may primarily reflect acute radiation exposure to peripheral blood during treatment, whereas the depth of the nadir may better capture the cumulative impact of therapy. When stratified by ALC nadir, significant differences in survival outcomes were observed in the BID group, but not in the QD group, highlighting the prognostic value of sustained immune suppression in this setting. Importantly, as no patients received consolidation immunotherapy in our study, the study did not evaluate the predictive value of ALC nadir for immunotherapy benefit. Nevertheless, prior reports have suggested that in patients receiving concurrent immunotherapy, a higher ALC nadir was significantly associated with prolonged PFS (20). The relatively higher ALC nadir observed in the BID group may indicate more favorable preservation of immune function. However, any potential implications for subsequent immunotherapy should be considered hypothesis-generating and require prospective validation.

EDRIC, a recently proposed model, quantifies the radiation dose to circulating immune cells during radiotherapy by integrating lung, heart, and body doses. Prior studies have shown that higher EDRIC is associated with more severe lymphopenia and poor survival outcomes (10, 17–19). We also observed a significant inverse correlation between ALC nadir and EDRIC (R = –0.44, *P* < 0.0001), indicating that greater radiation exposure may lead to deeper lymphocyte depletion. In this study, EDRIC was primarily used to evaluate prognostic stratification within each treatment group rather than as a direct quantitative comparator between fractionation schedules. When stratified by EDRIC, patients with an EDRIC ≤ 5.61 Gy had better survival outcomes compared with those with an EDRIC > 5.61 Gy in the BID group. In contrast, stratification by EDRIC did not result in significant differences in survival outcomes in the QD group, suggesting differential prognostic utility of this parameter according to fractionation schedule. EDRIC warrants consideration in radiotherapy planning to reduce lymphocyte exposure. Strategies aimed at minimizing radiation dose to circulating immune cells and immunologically relevant structures may help mitigate treatment-related lymphopenia. Proton therapy, for instance, can better preserve critical structures such as the thoracic vertebrae, major vessels, heart, and lungs, thereby reducing lymphocyte depletion (21–26). Optimizing dose distribution to immunologically relevant organs is anticipated to be a crucial focus in radiotherapy planning.

The underlying mechanisms for these differences may relate to variations in biological dose delivery, treatment time, and immune system recovery dynamics between fractionation regimens. Prior clinical studies have demonstrated that, compared with single high-dose radiotherapy (20 Gy×1), fractionated radiotherapy (3×8 Gy) more potently promotes the enrichment of CD8+ T cells around tumors and effectively mediates tumor regression (27). Conversely, high single-dose radiotherapy may upregulate 3’ endonuclease, leading to degradation of cytoplasmic DNA fragments and subsequent diminishment of immunogenic cell death effects (28). Compared with BID radiotherapy, the longer treatment duration and larger per-fraction dose in QD radiotherapy may result in more sustained and uniform immunosuppression, thereby reducing the discriminatory capacity of immune-related biomarkers. When immune injury reaches a relatively homogeneous or more severe level across patients, stratification based on ALC nadir or EDRIC may provide limited additional prognostic separation, suggesting that the prognostic utility of immune-related biomarkers may depend on the radiotherapy fractionation schedule.

These findings align with recent evidence from the ADRIATIC and NRG-LU005 studies. The results of the ADRIATIC study indicated that patients receiving BID radiotherapy derived greater benefit from consolidation durvalumab, showing superior OS compared with those receiving QD radiotherapy (3-year OS rate: 65.8% vs. 53.1) (4). Similarly, in the NRG-LU005 study, patients receiving BID radiotherapy had prolonged OS compared with those receiving QD radiotherapy (median OS: 35.4 vs. 28.3 months). These results suggest that BID radiotherapy may better preserve the immune microenvironment required for effective immunotherapy, potentially due to reduced immunotoxicity or more favorable dose distribution that spares immune-related structures. The choice of fractionation, therefore, may not only influence tumor control but also shape the immunologic landscape that determines the efficacy of subsequent immunotherapy.

Several limitations should be acknowledged. First, as a single-center retrospective analysis, selection bias cannot be excluded. Second, immune-related markers were limited to ALC nadir and EDRIC, without further analysis of lymphocyte subpopulations such as CD8^+^ T cells, regulatory T cells, or NK cells. Third, due to the retrospective design, lymphocyte measurements beyond the radiotherapy period were not uniformly available for all patients. Therefore, systematic evaluation of post-radiotherapy immune recovery could not be performed. Finally, the study did not directly assess outcomes after consolidation immunotherapy, so the impact of immune preservation on immunotherapy efficacy remains speculative. Prospective studies are warranted to validate these findings and explore the predictive utility of ALC nadir and EDRIC in the context of consolidation immunotherapy.

In conclusion, ALC nadir and EDRIC demonstrated prognostic significance in the BID group but not in the QD group, suggesting potential differences in radiation-associated immune effects between fractionation schedules. Further prospective studies are warranted to validate these observations.

## Data Availability

The raw data supporting the conclusions of this article will be made available by the authors, without undue reservation.

## Supplementary material

Supplementary Figure 1 Correlation analysis between EDRIC and ALC nadir. EDRIC, estimated dose of radiation to immune cells; ALC, Absolute lymphocyte count.

Supplementary Figure 2 Kaplan–Meier survival curves in the QD group stratified by QD-specific median values of ALC nadir and EDRIC. **(A, C, E)** Survival outcomes stratified by median ALC nadir: OS **(A)**, PFS **(C)**, and BMFS **(E)**. **(B, D, F)** Survival outcomes stratified by median EDRIC: OS **(B)**, PFS **(D)**, and BMFS **(F)**. ALC, Absolute lymphocyte count; OS, Overall survival; PFS, Progression-free survival; BMFS, Brain metastasis-free survival.
